# The Arabic Questionnaire for Psychotic Experiences in patients with psychotic disorders: a clinical validation

**DOI:** 10.1186/s12888-023-04649-4

**Published:** 2023-03-07

**Authors:** Arij Yehya, Salma M. Khaled, Iris E. C. Sommer, Saba F. Elhag, Mohamed H. M. O. Hassan, Peter Woodruff, Majid Alabdalla

**Affiliations:** 1 Core Curriculum Program, Deanship of General Studies, Qatar University, P.O. Box 2713, Doha, Qatar; 2 Social & Economic Survey Research Institute (SESRI), Qatar University, Doha, Qatar; 3 Department of Neuroscience and Department of Psychiatry, University Medical Center Groningen, Groningen, The Netherlands; 4 Psychiatry Department, Hamad Medical Corporation, Doha, Qatar; 5 Department of Neuroscience, School of Medicine, University of Sheffield, Sheffield, UK; 6 College of Medicine, Qatar University, Doha, Qatar

**Keywords:** Psychotic Disorders, Positive and Negative Syndrome scale, Questionnaire for Psychotic Experiences, Clinical Validity, Qatar

## Abstract

**Background:**

Psychotic experiences are reported in the general population. The Questionnaire for Psychotic Experiences (QPE) was created to test the phenomenological features of these experiences and compare them with those reported in patients with psychiatric and other medical conditions. The aim of this study was to test the psychometric properties of the Arabic version of the QPE.

**Methods:**

We recruited 50 patients with psychotic disorders from the Hamad Medical Hospital in Doha, Qatar. Patients underwent assessment over three sessions with trained interviewees using the Arabic versions of QPE, Positive and Negative Syndrome Scale (PANSS), Beck Depression Inventory (BDI), and the Global Assessment of Functioning Scale (GAF). Patients were also reassessed using the QPE and GAF after 14-days from the initial assessment in order to test for the stability of the scale. In this respect, this is the first study that assesses the test–retest reliability of the QPE. The psychometric properties including convergent validity, stability, and internal consistency met the benchmarked criteria.

**Results:**

Results confirmed that the Arabic version of QPE accurately measured the experiences of patients that were also reported using the PANSS, an internationally accepted, well-established scale for measuring psychotic symptom severity.

**Conclusion:**

We propose the use of the QPE to describe the phenomenology of PEs across modalities in Arabic speaking communities.

## Background

Psychotic experiences (PEs) [1] are common in many psychiatric disorders including schizophrenia, bipolar disorders, and schizoaffective disorders [2–4]. Patients with these disorders may experience any type of hallucination or delusions. These experiences have also been reported among individuals in the general population [5–10]. van Os, Linscott [8] noted that the prevalence of psychotic symptoms in the adult general population was 5.3%. Well-functioning individuals described experiencing hallucinations and delusions in several modalities. Another study showed that about 13.2% of the general public reported auditory hallucinations [11].

Many reliable and valid instruments are currently used to assess PEs [12]. However, each of the tools mentioned in the assessment review conducted by Hinterbuchinger and Mossaheb [12] poses it’s own limitations. For instance, the Psychosis Screening Questionnaire (PSQ) fails to provide a precise nature of the experiences [13]. Some questionnaires were constructed and validated in patients with psychotic disorders (such as schizophrenia). For instance, the Positive and Negative Syndrome scale (PANSS) developed by Kay [14] provides a general score for positive and negative symptoms rather than a descriptive one for certain delusions and specific types of hallucinations in different modalities. Another issue with structured interviews in general is that most of them need to be administer by trained personnel. For example, the Structured Interview for Prodromal Syndromes (SIPS) requires that the raters are well trained [15]. This would make data collection on population level challenging.

Rossell, Schutte [16] examined the reliability and validity of a new trans-diagnostic assessment tool for assessment of the phenomenology of PEs, the Questionnaire for Psychotic Experiences (QPE). A panel of experts developed 50-items that cover the following experiences: auditory and visual hallucinations, hallucinations in other modalities, and different types of delusions. Rossell, Schutte [16] provided evidence that the English version of the QPE had 100% accuracy for inter-rater reliability. The questionnaire could be easily administered without training. In addition, the subscales of this questionnaire had at least 70% test–retest accuracy within 7 days. The internal consistency of the scale had a Cronbach’s α > 0.7 for the subscales. The validity of the questionnaire was also evident as the subscales of the QPE correlated with several widely used measures including Positive and Negative Syndrome scale (PANSS). These psychometric properties were calculated after administering the QPE and other scales to patients with psychotic disorders as well as in non-clinical participants. Having this questionnaire translated and validated into different languages would provide a more informed understanding of cross-cultural PEs.

A study in the general population of Qatar reported that the lifetime prevalence of PEs was 27.9% [17]. These included visual hallucinations (12.8%), persecutory delusions (6.7%), and auditory hallucinations (6.9%). PEs were also more common among Arabs than non-Arabs. Therefore, there is a need for more research into the phenomenology of PEs using a valid and reliable transdiagnostic instrument in the Arabic language. Although the PANSS was translated and validated in the Arab clinical population in Qatar [18, 19], this semi-structured interview was aimed at patients with schizophrenia. Having a valid and reliable Arabic version of the QPE would allow evaluation of the prevalence and phenomenology of PEs across different medical conditions and psychiatric diagnoses in the general population. This would facilitate assessment of the predictors of developing PEs [10] and early detection of psychotic disorders [20]. However, the QPE is yet to be validated in Arabic-speaking clinical populations to date. This would be an important first step before use as a research tool in the general population or community-based settings.

“Prior to validating the tool among general population, we opted to test whether the QPE shows good construct validity. In other words, the questionnaire should be correlated with other gold standard questionnaires that assesses psychotic experiences. Patients with psychotic disorders experience PEs. Upon validation in this sample, we could move to validation among general population.” Accordingly, the aim of this study was to assess the reliability and the validity of the Arabic version of the QPE among patients with psychosis by comparing responses with a gold standard questionnaire (Arabic version of the PANSS; [18] within a sample of clinical patients with psychotic disorders. We aimed to determine the convergent validity, evaluated by comparing specific items pertaining to hallucinations and delusions from the QPE with the PANSS in a sample of patients diagnosed with a variety of psychotic disorders. Rossell and Schutte [16] showed that the QPE was uncorrelated to BDI; hence we opted to use it to test for discriminant validity. We also wished to ascertain the discriminant validity, evaluated by comparing the scores on the QPE with the Beck Depression Inventory [21, 22]. Finally, we aimed to evaluate the test–retest reliability of the QPE in a subset of our sample.

## Methods

This paper covers the clinical validation of the QPE questionnaire among patients with psychiatric disorders for understanding the phenomenology of psychotic experiences in clinical in Qatar (http://pe-qatar.com/).

### Measures

#### Questionnaire for Psychotic Experiences

The QPE assessed the presence and the phenomenological characteristics pertaining to various forms of PEs. In addition to lifetime and current prevalence of PEs, the 50-items in the entire scale could be grouped into four different subscales: auditory hallucinations, visual hallucinations, hallucinations in other modalities, and delusions. Current prevalence of PEs are assessed during the past week in reference to the interview date. For the prevalence of the each PEs, participants answered a Yes/No question first to evaluate whether they had such experiences, upon answering “Yes” they were asked further questions on the features of the specific PEs. For the purpose of this study, the scores of the entire QPE and each subscale were computed by summing up the 6-level (0–5) responses to items for measuring frequency, duration, distress and impact of these phenomena.

Before translation to Arabic, we adapted the English version of the QPE for self-administration by removing open-ended probes. In addition, in the delusions section, the items for frequency, duration, distress and impact were asked once for all the different types of delusions. This shortened the length of the QPE in an effort to make the response rate higher when used on a wide scale in the population. We could assess the features of delusions by association to the type of delusion the participants recorded during the past week; hence, this would increase response rate as the QPE is less time consuming to fill and we could retain key features for delusions. The QPE was translated into formal Arabic using the back translation method. In brief, after the initial translation, a team of experts on psychometrics in psychiatry/psychology reviewed and amended the questionnaire. An independent team of bilingual researchers then translated the updated version back to English and the main authors of the English version of the QPE reviewed and approved this version. The mean length of the QPE in minutes during the first administration was 19.18 (SD = 15.04).

#### PANSS

The Positive and Negative Syndrome Scale (PANSS) was developed by [14]. It was translated and validated in Arabic patients with schizophrenia in Qatar [18, 19]. The PANSS rating was based on the Structured Clinical Interview for PANSS (SCI-PANSS) that was also translated to Arabic. This interview took between 30 and 45 min to administer. All interviewers were trained to conduct the PANSS interview, which is composed of three subscales: Positive, Negative and General Psychopathology. Each item within the subscale is reported from 1 to 7, ranging from absent to extreme.

#### Beck Depression Inventory

The Beck Depression Inventory (BDI) was developed by [21]. It comprises 21 items each with four choices. This self-rating scale was translated to Arabic by [22]. The Arabic version of the scale has good internal consistency [22].

#### GAF

The Global Assessment of Functioning (GAF) scale was used to assess the social, occupational, and psychological functioning of the patients [23]. It is a 100-point-rating scale with a higher score representing a better functioning state. The scale measures the change in impairment caused by mental illness over time. The English version of the scale was used given that all the trained interviewers were bilingual.

### Participants

The study was approved by the Institutional Review Board at Hamad Medical Corporation approved the study on May 26^th^, 2021 (QU-IRB 1021-EA/19).We then selected a sample of 50 adult patients clinically diagnosed with psychotic disorder from various mental health services in Hamad Medical Cooperation, Doha, Qatar. Consented participants included those from inpatient units, community outreach health care centers and outpatient clinics based Psychiatry Hospital, Community Services in Muaither, Al Wakra Hospital and Rumailah Hospital. These services are widespread across Qatar making our sample more geographically representative of patients’ severity from many areas of the country.

### Procedure

**Fig. 1:**
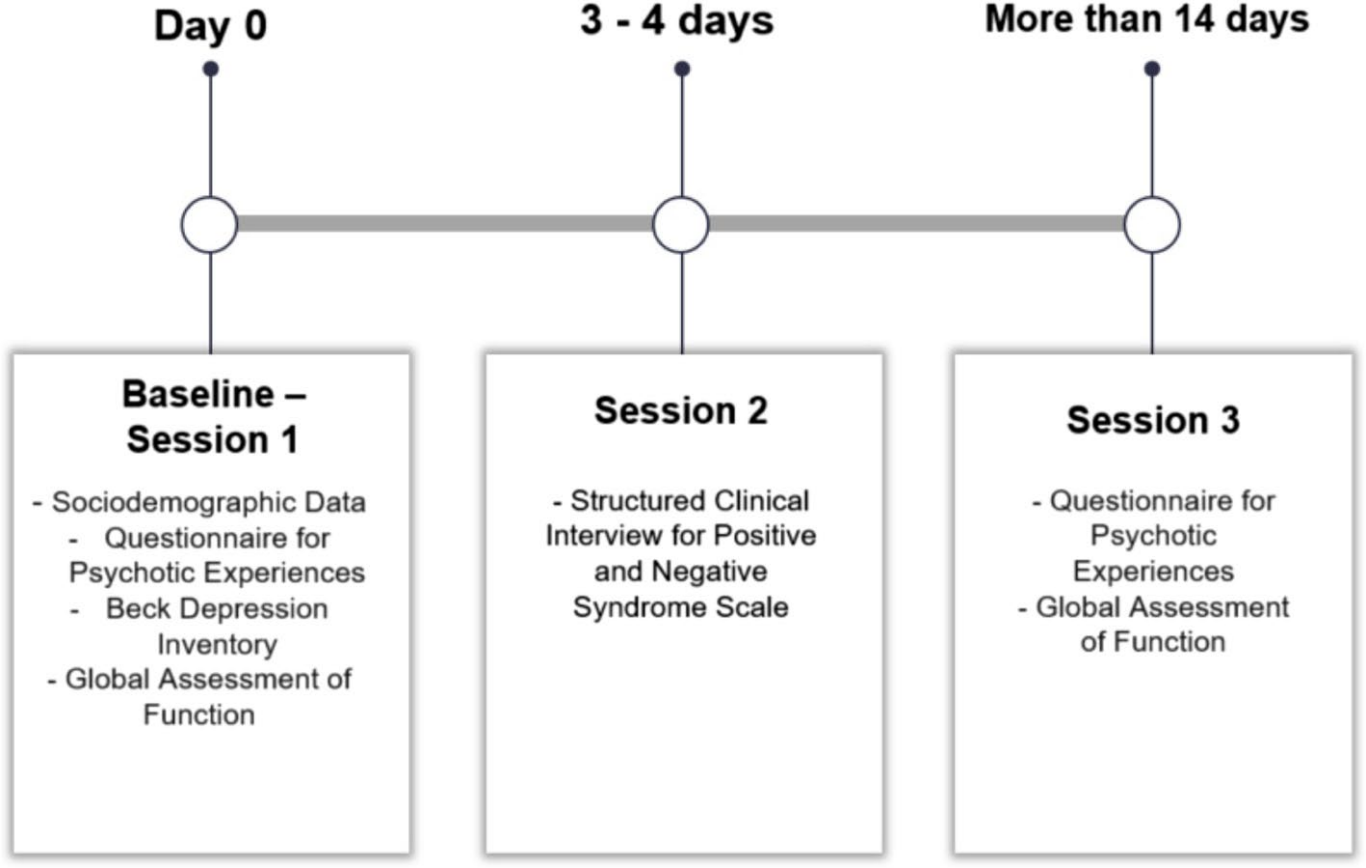
The administered scales during the three assessment sessions

Three raters administered all the measures. All raters were trained to administer the SCI-PANSS interview and had a medical degree or Masters level degree in psychology. Each rater administered the questionnaire to 15 patients. Inter-rater reliability was calculated for a total of 5 patients for all the scales. These patients completed three face-to-face sessions with the rater, each lasted approximately 45 min. In the first session, the QPE, and the Beck depression inventory was administered. In addition, sociodemographic data were collected from the patient. Based on the assessment session and healthcare provider reports, the raters logged in the GAF score of the patient. In the second session, the rater administered the structured interview of the PANSS. The second session was conducted between 3 to 4 days after the first session. The third session took place after 14 days from the baseline, during which the QPE and GAF were administered again. Additional data including number of hospital admissions, psychiatrist clinical diagnosis, age of onset, medications and medication doses were extracted from the informational system of the hospital. Figure 1 illustrates the data collection procedures of the study.

### Statistical analysis

Patient characteristics were presented as mean and standard deviation for age, score on QPE subscales, score on PANSS, BDI, GAF, and medication doses. In addition, variables including gender, nationality, recruitment center, marital status, diagnosis, and the type of antipsychotics were presented in frequencies. The prevalence of the different types of delusions and hallucinations were reported as current and lifetime estimates.

To assess the psychometric properties of the Arabic version of the QPE, first convergent validity was assessed by calculating Kendall’s tau correlation coefficients due to non-normality [24] between specific items on the PANSS (related to hallucinations and delusions) and the QPE subscales. A value greater than 0.40 is considered moderate correlation [25]. In addition, the QPE scores were correlated with the GAF scores using Kendall’s tau correlation coefficients. Second, divergent validity was calculated by comparing the scores on QPE with the PANSS negative and general ratings and scores on the BDI. Third, internal consistency was calculated using Cronbach’s α for each of the subscales of the QPE. A coefficient with values more than 0.7 were considered acceptable [26, 27]. Fourth, the Cronbach’s α was used to calculate inter-item correlation for QPE items. Fifth, test–retest analysis was conducted by correlating scores on the QPE subscales for the two sessions conducted more than 14 days apart using the Kendall’s tau correlation and intraclass-correlation coefficients [28]. Finally, ICC were also used to compare inter-rater reliability among the raters **on all the scales** for the 5 patients. The cut-off of 0.40 is considered fair for ICC values, above 0.60 is good are having values more than 0.75 is considered excellent [28].

## Results

### Participants

Table 1 shows the sociodemographic, clinical characteristics and prescribed treatment for the sample of 50 Arab patients. The mean age was 36.7 years (SD = 12.6), 16.0% were female and 52.0% were Qataris. Most of them were never married and the majority had university postgraduate or secondary high school education.

**Table 1:** Sociodemographics, clinical characteristics and treatment of the sample of 50 patients

	Variable	Frequency (%)
**Gender**	Male	42 (84%)
	Female	8 (16%)
**Nationality**	Qatari	26 (52%)
	Non-Qatari Arabs	24 (48%)
**Marital Status**	Married	14 (28%)
	Divorced	5 (10%)
	Never Married	31 (62%)
**Education**	Primary School	9 (18%)
	Secondary School/ High School	20 (40%)
	University / Postgraduate	21 (42%)
**Subsample**	Outpatient	12 (24%)
	Community	16 (32%)
	Inpatient & Long term Residential	22 (44%)
**Diagnosis**	Schizophrenia	36 (72%)
	Acute Psychotic Episode	2 (4%)
	Schizoaffective	5 (10%)
	Bipolar with Psychotic experiences / Bipolar with Schizophrenia	6 (12%)
	Substance induced Psychosis	1 (2%)
**Antipsychotics**	First generation antipsychotics (FGA)	10 (2%)
	Second generation antipsychotics (SGA)	33 (66%)
	Combination of FGA & SGA	3 (6%)
	None	1 (2%)
	Missing information	3 (6%)

Sociodemographic and diagnostic information of patients are also shown in Table 1. All but one patient received antipsychotic medication. The mean age of onset was 26.1 (SD = 8.5) and the mean number of hospitalizations was 3.4 (SD = 5.2).

The completion rate for the QPE and BDI was 100%. For the PANSS, the completion rate was 46 out of the 50 (92%).

Table 2 presents the clinical profile of the sample based on the scales assessed in this study. The lifetime prevalence of auditory hallucinations in our sample was 68% and the lifetime prevalence of delusions was 72%. Current auditory hallucinations and delusions were of similar in prevalence as were visual and tactile hallucinations. Olfactory hallucinations were the least common type of hallucinations. Only 18% of the sample experienced multimodal hallucinations.

**Table 2:** Clinical characteristics based on questionnaires and scales administered

		**Frequency (%)**		**Mean**
**QPE**		**L**	**C**	
**Hallucinations**				35 (9.3)
	Auditory Hallucinations	34 (68%)	14 (28%)	
	Visual Hallucinations	16 (32%)	3(6%)	
	Tactile Hallucinations	15 (30%)	5(10%)	
	Olfactory Hallucinations	11 (22%)	0 (0%)	
	Sensed Hallucinations	15 (30%)	9 (18%)	
	Multimodal Hallucinations		9 (18%)	
**Delusions**		36 (72%)	17 (34%)	5.5(6.6)
**PANSS**	Positive Scale			16.5 (6.9)
	Negative Scale			14.8 (8.0)
	General Scale			30.5 (11.4)
	Total Score			61.8 (21.9)
**BDI**	Low	31 (62%)		38.0(10.1)
	Moderate	9 (18%)		
	High	10 (20%)		
**GAF**	1–30	11 (22%)		58.8(28.0)
	31 – 60	15 (30%)		
	61 – 80	11 (22%)		
	81 -100	13 (26%)		

*Abbreviations*: *QPE* Questionnaire for Psychotic Experiences, *L* Lifetime prevalence, *C* Current prevalence, *PANSS* Positive and Negative Syndrome Scale, *BDI* Beck Depression Inventory, *GAF* Global Assessment of Function, *%* Percentage of the total number of patients, *SD* Standard deviation

Delusions were more prevalent than hallucinations in this sample, with 72% lifetime and 34% current experiences of different types of delusions. Among the different types of delusions, paranoid delusions were most common (29 cases, 58%), while reference, guilt, control and grandeur delusions all were similarly prevalent (between 28 to 32%). The least common type of delusions experienced among the sample was somatic (only 5 cases or approximately 10%). According to the GAF scores, the patients had a wide range when it comes to severity and social functioning. The mean of the total score for the PANSS was 61.8 (SD = 21.9).

QPE provided further details about the distress, duration, type and impact of PEs experienced by our participants. These were only for those that happened within the last week (i.e. current). For auditory hallucinations, these lasted for around minutes but less than an hour for most of the participants who experienced them (12 out of 14, 85.7%). Most of them also reported that these experiences had an impact on of their daily activities (9 out of 14, 64.3%). All but one participant reported that visual hallucinations lasted for an hour or less (5 out of 6, 83.3%). In addition, three of them (50%) mentioned that they had an impact on their daily activities. As for the delusions, the impact on the daily activities was for 64.7% of participants who experienced them during the last week (11 participants).

### Validity

Table 3 provides the convergent and divergent validity of the QPE subscales (lifetime and current prevalence estimates). Lifetime and current visual and auditory hallucinations QPE subscales significantly correlated with the PANSS hallucinations subscales (*p* < 0.05). The QPE delusions subscales significantly correlated with the PANSS subscale for delusions confirming validity of this subscale as per the pre-defined criteria [24, 25]. The GAF negatively correlated with lifetime and current experiences of delusions and lifetime experiences of auditory hallucinations as assessed by the QPE.

**Table 3:** Convergent and divergent validity of QPE subscales

		**QPE Subscales**											
		**AH**				**VH**				**D**			
		**L**		**C**		**L**		**C**		**L**		**C**	
		**Kendall’s tau**	***p*-value**	**Kendall’s tau**	***p*-value**	**Kendall’s tau**	***p*-value**	**Kendall’s tau**	***p*-value**	**Kendall’s tau**	***p*-value**	**Kendall’s tau**	***p*-value**
**Convergent Validity**													
PANSS	Hallucinations	**0.45**	< 0.0001	0.52	**0.002**	**.45**	< 0.0001	**0.58**	0.026	**0.34**	< 0.0001	-0.05	0.710
	Delusions	**0.32**	**0.015**	0.17	0.027	**0.29**	0.027	0.33	**0.203**	**0.38**	0.001	**0.53**	< 0.0001
GAF		**-0.34**	**0.004**	-0.03	0.849	-0.23	0.059	-0.41	0.064	**-0.32**	0.003	**-0.47**	< 0.0001
**Divergent Validity**													
PANSS	Negative	0.12	0.326	0.25	0.105	0.03	0.845	0.29	0.245	0.02	0.829	0.02	.885
	General	0.21	0.085	-0.11	0.480	0.11	0.373	0.39	0.121	**0.29**	0.001	**-0.40**	.002
BDI		**-0.35**	**0.004**	0.52	0.849	0.18	0.132	0.31	0.157	**0.48**	< 0.0001	**-0.34**	.006

The table presents the Kendall’s tau correlation coefficients between the different subscales of QPE and the administered questionnaires

*Abbreviations*: *QPE* Questionnaire for Psychotic Experiences, *AH* Auditory hallucinations, *VH* Visual hallucinations, *D* Delusions, *L* Lifetime prevalence, *C* Current prevalence, *PANSS* Positive and Negative Syndrome Scale, *BDI* Beck Depression Inventory, *GAF* Global Assessment of Function, *BDI* Beck Depression Inventory, *N* = 46

As shown in Table 3, tests of divergent validity showed that there were no significant correlations between the PANSS negative and general subscales and the QPE subscales for hallucinations (*p* > 0.05). However, the only significant correlations were between lifetime and current QPE delusions and the general PANSS subscales. The correlation was a negative one between PANSS general score and current delusions. The BDI significantly correlated with lifetime experiences of auditory hallucinations and current and lifetime experiences of delusions. The BDI score also significantly correlated with delusions score on the PANSS, *r*(46) = 0.52, *p* < *0.0001*.

### Reliability

The internal consistency of the QPE was calculated for the different items within the same subscale. The subscales had a Cronbach alpha of 0.75 for auditory hallucinations, 0.74 for visual hallucinations and 0.79 for delusions. Interrater reliability on the QPE scale had an intra-class correlation between 0.83 – 1.00 on the five different cases meaning that the interrater reliability was good for QPE. For the scales PANSS, BDI and GAF the interrater reliability was also good with ICC score between 0.81 – 0.97. Test–retest reliability showed consistency greater than 0.50 across time. This was for all the lifetime subscales (*p* < 0.01). However, none of the 37 patients reported any current experiences of visual or olfactory hallucinations in the third session. More so, current tactile hallucinations were inconsistent after 2 weeks. Table 4 shows the results of test–retest reliability.

**Table 4:** Test–retest Reliability: QPE Subscales administered more than 14 days apart

QPE Subscales		Frequency in the 2^nd^ session	Frequency in the 3^rd^ session	Kendall’s Tau correlation	*p*-value	Interclass Correlation ICC[95%CI]	*p*-value
**Auditory Hallucinations**	**L**	25 (67.6%)	21 (56.8%)	**0.68**	< 0.0001	**0.81** [.63-.90]	< 0.0001
	**C**	10 (27%)	10(27%)	**0.60**	< 0.0001	**0.84**[.69-.92]	< 0.0001
**Visual Hallucinations**	**L**	11 (29.7%)	8 (21.6%)	**0.52**	0.002	**0.68**[.38-.84]	< 0.0001
	**C**	1 (2.7%)	0(0%)	-	-	-	-
**Tactile Hallucinations**	**L**	12 (32.4%)	9 (24.3%)	**0.56**	0.000	**0.71**[.43 -.85]	< 0.0001
	**C**	3 (8.1%)	2(5.4%)	0.91	0.823	**0.53**[.09-.76]	0.013
**Olfactory Hallucinations**	**L**	8 (21.6%)	6 (16.2%)	**0.84**	< 0.0001	**0.91**[.83-.95]	< 0.0001
	**C**	0(0%)	0(0%)	-	-	-	-
**Sensed Hallucinations**	**L**	11 (29.7%)	8 (21.6%)	**0.81**	< 0.0001	**0.89**[0.79–0.94]	< 0.0001
	**C**	6 (16.2%)	3(8.1%)	**0.68**	< 0.0001	**0.79**[0.58–0.89]	< 0.0001
**Delusions**	**L**	26(70.3%)	21(56.8%)	**0.77**	< 0.0001	**0.85**[0.71–0.92]	< 0.0001
	**C**	11(29.7%)	11(29.7%)	**0.61**	< 0.0001	**0.76**[0.53–0.88]	< 0.0001
**GAF Score (M(SD))**		64.65 (24.99)	71.28 (21.02)	**0.58**	< 0.0001	**0.73**[0.47–0.86]	< 0.0001

*Abbreviations*: *QPE* Questionnaire for Psychotic Experiences, *L* Lifetime prevalence, *C* Current prevalence, *GAF* Global Assessment of Function*, M* Mean, *SD* Standard deviation,* N* = *37*

## Discussion

The aim of this study was to validate the Arabic version of the QPE among a sample of patients with psychotic disorders. In selecting patients with a range of psychotic disorders from various health centers in Qatar, we anticipate that our results to be representative of patients who use outpatient services for severe psychiatric conditions in Qatar. The completion rate for the two assessment sessions was 92%, which was high given the length of each session (about 45 min). In addition, inter-rater reliability on the QPE and internal consistency of this scale was equivalent to the reliability nalysis in the English version of the QPE [16].

Among our sample of patients, we detected high frequency of lifetime delusions and auditory hallucinations, reaching more than 67%. In addition, lifetime prevalence of visual, sensed, tactile and olfactory hallucinations were relatively high (minimum of 22.0%). The lower prevalence of current experiences of hallucinations and delusions (34%) in our sample may reflect their varied diagnostic nature and low symptom severity as measured by the GAF and PANSS.

The validity of the QPE was established when compared with the PANSS subscales for hallucinations and delusions. Our hypothesis regarding the divergent validity of the QPE scale with PANSS negative general psychopathology subscale along with BDI was confirmed for both auditory and visual hallucinations. Having lifetime and/or current delusions on the QPE was associated with higher severity of global functioning as measured by the GAF, General Psychopathology and the BDI scales. This finding was also confirmed by the significant correlation between the PANSS delusions and the BDI scale. Previous research showed that indeed patients with higher depression presented with more severe delusions [29]. The results also showed that lifetime delusions were positivity correlated with PANSS General Psychopathology; however, it was negatively correlated with having current delusions. One explanation is that the scales maybe measuring different things- and there is a third factor not being accounted. Another explanation is that the current assessments have different sensitivity to the same item from that measured over the lifetime.

This study is the first to assess the test–retest reliability of the QPE showing that indeed the scale measurements remained consistent after more than 14 days from the last assessment. All the lifetime assessments of PEs correlated across time. However, current experiences from the QPE were only consistent over time for auditory, sensed hallucinations and delusions. This outcome was expected as these current experiences were evaluated for the past week, while the test–retest administration had a gap of at least 14 days. All but one patient were on antipsychotics which may explain why the symptoms got better with time. Since a large portion of the participants were inpatients, this might explain why patients got better with time as they were given medications (antipsychotics – all but one patient) or have their medication changed because of presenting with symptoms that require inpatient care. The scores on GAF show that, in general, the patients increased with time.

The QPE captures a wide range of PEs that are phenomenological in nature including the type, severity, distress, and impact caused by those experiences. Subtyping PEs across different clinical diagnoses provides insight into the underlying mechanisms of these phenomena [30]. In this study, we used the QPE to report on the types of delusions and hallucinations of patients with PEs in the Qatari community.

### Limitations

 There are some limitations to this study. First, the number of females in this sample was low, and given the gender differences in the phenomenology [31], we need to generalize our results with caution. Second, specific delusions were correlated with higher depression scores in our sample. This limited our assessment of divergent validity. Nevertheless, there was no association between QPE subscales and PANSS negative and general subscale. The only association was between PANSS general subscale and delusions. Third, the sample size for the inter-rater reliability was small and hence generalizability of our findings is limited. Whilst a strength of our study was to provide information about the validity and reliability of the Arabic version of QPE among patients with psychosis, further investigation of its validity is needed among the general population. Fourth, further information on lifetime and current experiences was also assessed using this questionnaire, which were lacking in the Arabic version of the PANSS (reporting only the current experiences) [18]. Fifth, within the sample, there were low prevalence of some psychotic experiences such as olfactory and tactile hallucinations; hence further investigations to the reliability of these PEs within a different sample.

## Conclusion

The current data provide evidence that the Arabic version of the QPE is reliable with evidence for its convergent validity relative to a gold standard instrument like the PANSS. In this study, we demonstrated that the Arabic version of the QPE was measuring various forms of PEs and their severity consistently in our sample. The consistency of the scale was also demonstrated across time was also supported in our results for the lifetime prevalence of these experiences. The Arabic version of the QPE provided rich data on the prevalence and the phenomenology of these unusual experiences paving the way to a better understanding of psychosis. In addition, the English QPE is also valid within control patients [16] and was designed for use among different sub-populations. With evidence of the clinical validity of the Arabic version of the QPE, researchers can use this assessment tool in the general population or community-based settings to learn more about the experiences and their relation to other environmental factors such as religiosity and cultural beliefs.

## Data Availability

The datasets used and/or analysed during the current study are available from the corresponding author on reasonable request.

## Abbreviations

BDI: Beck Depression Inventory
GAF: Global Assessment of Functioning
ICC: Interclass Correlation
PANSS: Positive and Negative Syndrome Scale
PE: Psychotic experiences
QPE: Questionnaire for Psychotic Experiences
SCI-PANSS: Structured Clinical Interview for PANSS
SD: Standard Deviation

## References

[1] Clemmensen L, van Os J, Drukker M, Munkholm A, Rimvall MK, Vaever M (2016). Psychotic experiences and hyper-theory-of-mind in preadolescence–a birth cohort study. Psychol Med.

[2] Merrett Z, Rossell SL, Castle DJ (2016). Comparing the experience of voices in borderline personality disorder with the experience of voices in a psychotic disorder: A systematic review. Aust N Z J Psychiatry.

[3] Toh WL, Thomas N, Rossell SL (2015). Auditory verbal hallucinations in bipolar disorder (BD) and major depressive disorder (MDD): A systematic review. J Affect Disord.

[4] Bebbington P, Freeman D (2017). Transdiagnostic Extension of Delusions: Schizophrenia and Beyond. Schizophr Bull.

[5] Sommer IE, Daalman K, Rietkerk T, Diederen KM, Bakker S, Wijkstra J (2010). Healthy individuals with auditory verbal hallucinations; who are they? Psychiatric assessments of a selected sample of 103 subjects. Schizophr Bull.

[6] Maijer K, Begemann MJH, Palmen S, Leucht S, Sommer IEC (2018). Auditory hallucinations across the lifespan: a systematic review and meta-analysis. Psychol Med.

[7] Kelleher I, Cannon M (2011). Psychotic-like experiences in the general population: characterizing a high-risk group for psychosis. Psychol Med.

[8] van Os J, Linscott RJ, Myin-Germeys I, Delespaul P, Krabbendam L (2009). A systematic review and meta-analysis of the psychosis continuum: evidence for a psychosis proneness-persistence-impairment model of psychotic disorder. Psychol Med.

[9] Yung AR, Nelson B, Baker K, Buckby JA, Baksheev G, Cosgrave EM (2009). Psychotic-like experiences in a community sample of adolescents: implications for the continuum model of psychosis and prediction of schizophrenia. Aust N Z J Psychiatry.

[10] McGrath JJ, Saha S, Al-Hamzawi A, Alonso J, Bromet EJ, Bruffaerts R (2015). Psychotic Experiences in the General Population: A Cross-National Analysis Based on 31,261 Respondents From 18 Countries. JAMA Psychiat.

[11] Beavan V, Read J, Cartwright C (2011). The prevalence of voice-hearers in the general population: a literature review. J Ment Health.

[12] Hinterbuchinger B, Mossaheb N. Psychotic-Like Experiences: A Challenge in Definition and Assessment. Front Psychiatry. 2021;12:582392. 10.3389/fpsyt.2021.582392. 10.3389/fpsyt.2021.582392 PMC8039445 33854445

[13] Bebbington P, Nayani T. The psychosis screening questionnaire. Int J Methods Psychiatr Res. 1995;5(1):11–19.

[14] Kay SR. Positive and negative syndromes in schizophrenia: assessment and research. Brunner/Mazel; 1991.

[15] Miller TJ, McGlashan TH, Rosen JL, Cadenhead K, Cannon T, Ventura J (2003). Prodromal assessment with the structured interview for prodromal syndromes and the scale of prodromal symptoms: predictive validity, interrater reliability, and training to reliability. Schizophr Bull.

[16] Rossell SL, Schutte MJL, Toh WL, Thomas N, Strauss C, Linszen MMJ (2019). The Questionnaire for Psychotic Experiences: An Examination of the Validity and Reliability. Schizophr Bull.

[17] Khaled SM, Wilkins SS, Woodruff P (2020). Lifetime prevalence and potential determinants of psychotic experiences in the general population of Qatar. Psychol Med.

[18] Yehya A, Ghuloum S, Mahfoud Z, Opler M, Khan A, Hammoudeh S (2016). Validity and Reliability of the Arabic Version of the Positive and Negative Syndrome Scale. Psychopathology.

[19] Yehya A, Ghuloum S, Mahfoud Z, Opler M, Khan A, Hammoudeh S (2017). Validation of the Five-Factor Model of the Arabic Version of the Positive and Negative Syndrome Scale in Schizophrenia. Psychopathology.

[20] Fusar-Poli P, Bonoldi I, Yung AR, Borgwardt S, Kempton MJ, Valmaggia L (2012). Predicting psychosis: meta-analysis of transition outcomes in individuals at high clinical risk. Arch Gen Psychiatry.

[21] Beck AT, Steer RA (1984). Internal consistencies of the original and revised Beck Depression Inventory. J Clin Psychol.

[22] Abdel-Khalek AM (1998). Internal consistency of an Arabic adaptation of the Beck Depression Inventory in four Arab countries. Psychol Rep.

[23] Pedersen G, Karterud S (2012). The symptom and function dimensions of the Global Assessment of Functioning (GAF) scale. Compr Psychiatry.

[24] Akoglu H (2018). User's guide to correlation coefficients. Turkish J Emerg Med.

[25] Dancey CP, Reidy J (2007). Statistics without maths for psychology: Pearson education.

[26] Bland JM, Altman DG (1997). Statistics notes: Cronbach's alpha. Bmj.

[27] Terwee CB, Bot SD, de Boer MR, van der Windt DA, Knol DL, Dekker J (2007). Quality criteria were proposed for measurement properties of health status questionnaires. J Clin Epidemiol.

[28] Cicchetti DV (1994). Guidelines, criteria, and rules of thumb for evaluating normed and standardized assessment instruments in psychology. Psychol Assess.

[29] Gournellis R, Tournikioti K, Touloumi G, Thomadakis C, Michalopoulou PG, Michopoulos I (2018). Psychotic (delusional) depression and completed suicide: a systematic review and meta-analysis. Ann Gen Psychiatry.

[30] McCarthy-Jones S, Thomas N, Strauss C, Dodgson G, Jones N, Woods A (2014). Better than mermaids and stray dogs? Subtyping auditory verbal hallucinations and its implications for research and practice. Schizophr Bull.

[31] Esterberg ML, Trotman HD, Holtzman C, Compton MT, Walker EF (2010). The impact of a family history of psychosis on age-at-onset and positive and negative symptoms of schizophrenia: A meta-analysis. Schizophr Res.

